# Direct Observation of α-Synuclein Amyloid Aggregates in Endocytic Vesicles of Neuroblastoma Cells

**DOI:** 10.1371/journal.pone.0153020

**Published:** 2016-04-22

**Authors:** Mihaela M. Apetri, Rolf Harkes, Vinod Subramaniam, Gerard W. Canters, Thomas Schmidt, Thijs J. Aartsma

**Affiliations:** 1 Huygens-Kamerlingh Onnes Laboratory, Leiden University, Leiden, The Netherlands; 2 FOM Institute AMOLF, Amsterdam, The Netherlands; Louisiana State University Health Sciences Center, UNITED STATES

## Abstract

Aggregation of α-synuclein has been linked to both familial and sporadic Parkinson’s disease. Recent studies suggest that α-synuclein aggregates may spread from cell to cell and raise questions about the propagation of neurodegeneration. While continuous progress has been made characterizing α-synuclein aggregates *in vitro*, there is a lack of information regarding the structure of these species inside the cells. Here, we use confocal fluorescence microscopy in combination with direct stochastic optical reconstruction microscopy, dSTORM, to investigate α-synuclein uptake when added exogenously to SH-SY5Y neuroblastoma cells, and to probe *in situ* morphological features of α-synuclein aggregates with near nanometer resolution. We demonstrate that using dSTORM, it is possible to follow noninvasively the uptake of extracellularly added α-synuclein aggregates by the cells. Once the aggregates are internalized, they move through the endosomal pathway and accumulate in lysosomes to be degraded. Our dSTORM data show that α-synuclein aggregates remain assembled after internalization and they are shortened as they move through the endosomal pathway. No further aggregation was observed inside the lysosomes as speculated in the literature, nor in the cytoplasm of the cells. Our study thus highlights the super-resolution capability of dSTORM to follow directly the endocytotic uptake of extracellularly added amyloid aggregates and to probe the morphology of *in situ* protein aggregates even when they accumulate in small vesicular compartments.

## Introduction

Progressive accumulation and deposition of specific protein aggregates is a characteristic of many neurodegenerative disorders, including Parkinson’s disease (PD). In PD, α-synuclein(α-syn), a small presynaptic protein (~15 kDa), is the main fibrillar component of the intraneuronal protein aggregates (Lewy bodies) that represent the pathological feature of this disease [1]. Although α-syn is predominantly a cytosolic protein, recent studies suggest the protein exerts not only a pathogenic effect inside the cells, but an extracellular pathogenic action as well. Multiple forms of α-syn have been observed in cerebrospinal fluid, blood plasma and more recently, in saliva [2–4]. When applied to cultured cells, α-syn preformed aggregates are internalized via endocytosis and targeted to the lysosomes for degradation [5–9]. The extent of aggregate accumulation inside cells is determined by the cells ability to degrade and remove the aggregates. Few groups reported that α-syn taken-up from the extracellular space induces the aggregation of the endogenous protein leading to the formation of Lewy body-like inclusions [9–14]. Cell to cell transmission of α-syn pathological aggregates most likely through sequential exocytosis and endocytosis, has been demonstrated in neuronal cultured cells as well as in animal models [15, 16].

At this moment, the fate of the exogeneous α-syn aggregates once they enter the cells is not clear. Are the aggregates degraded in the lysosomes, or do they start growing into larger α-syn aggregates? Do they overload the degradation systems impairing their activity and escape in the cytosol inducing aggregation of the endogeneous protein? In order to address these questions, we followed directly the uptake and fate of α-syn preformed aggregates when added to neuroblastoma cells, SH-SY5Y, by super-resolution microscopy.

While atomic force microscopy (AFM) has been extensively used to obtain the ultrastructure and morphological features of the amyloid aggregates, the technique has the drawback to be applicable only *ex situ*. In contrast, optical microscopy and in particular the new super-resolution methods are powerful and non-invasive techniques for the study of morphological features of the amyloid aggregates with nanometer resolution. In the last years, these optical techniques have been successfully used in several studies to probe the morphology of protein aggregates *in vitro* [17–19] and in cells [20–22].

Here we applied confocal fluorescence microscopy and optical super-resolution microscopy to follow and characterize the fate of small *in vitro* assembled α-syn fibrils in human neuroblastoma cells. We found that fibrils were shortened when trafficked through the lysosomal pathway. Further fibril maturation and formation of long fibrils was not observed. Our study thus highlights the potential role of lysosomal degradation in the prevention of α-syn aggregation in cells.

## Materials and Methods

### Preparation of labeled α-syn fibrillar seeds

Recombinant human wild type α-syn was expressed and purified as described previously [23]. Fibrils were formed at 37°C in 1.5 ml Eppendorf tubes under constant agitation (1000 rpm, in an Eppendorf Thermomixer comfort, Eppendorf AG, Germany). 300 μL of 70 μM α-syn in phosphate buffer saline (PBS) was incubated for 5 days. We stopped the aggregation process when the plateau was reached as seen by thioflavin T fluorimetry (data not shown). The presence of fibrils was confirmed by atomic force microscopy. Labeling of α-syn fibrils with the NHS ester (succinimidyl ester) of Alexa Fluor 532 and Alexa Fluor 647 was performed according to the manufacturer’s instructions (Life Technologies, USA). Briefly, α-syn fibril solution was incubated for 1 h at room temperature with Alexa Fluor 532 dye in a 1:1 protein/fluorophore molar ratio. The unbound dye was removed by pelleting the fibrils at 13000 rpm for 15 min in a tabletop centrifuge. The supernatant was discarded and the pellet containing labeled fibrils was resuspended in PBS. The centrifugation/resuspension cycle was repeated twice. Purified labeled α-syn fibrils were divided in 20 μL aliquots, flash frozen and stored at -80°C. Fibrillar seeds of α-syn were produced as following: 20 μL of labeled fibrils were diluted 10 times in PBS and sonicated 3×5 s with a probe sonicator (Sonics & Materials, Inc., USA) using 50% maximum power, yielding variable fibril lengths between 50 to 700 nm. These seeds were added immediately in the culture medium of SH-SY5Y cells at a final concentration of 100 nM, and their uptake by the cells was followed in time using confocal microscopy and direct stochastic optical reconstruction microscopy (dSTORM).

### Cell culture

Human neuroblastoma cells, SH-SY5Y (ATCC), were grown in 1:1 minimum essential media (MEM) (Gibco by Life Technologies, USA) and nutrient mixture Ham’s F-12 (PAN Biotech, Germany) free of phenol red, supplemented with 1% MEM, non-essential amino acids, 2mM glutamax and 15% fetal bovine serum (Gibco by Life Technologies).

### Atomic Force Microscopy (AFM)

Labeled α-syn fibrillar samples were diluted 5 times into PBS, and 10 μL were pipetted onto freshly cleaved mica and kept at room temperature for 60 s. The mica surface was then rinsed with Millipore-filtered water (2×50 03BCL) to remove loosely bound protein, dried under a stream of nitrogen and imaged immediately. AFM imaging was performed on a MultiMode Nanoscope IIIa microscope (Digital Instruments, USA) equipped with an E-scanner. All measurements were carried out in the tapping mode under ambient conditions using single-beam silicon cantilever probes with a resonant frequency of 300 kHz (Olympus, Japan). Image analysis was performed using the instrument software.

### Co-localization experiments with lysosomes

SH-SY5Y cells were incubated with 50 nM LysoTracker® Deep Red (Life Technologies) for 30 min at 37°C, then washed and incubated further with 100 nM Alexa532-labeled α-syn sonicated fibrils. For colocalization experiments with CellLight® Lysosomes-RFP, BacMam 2.0 (Life Technologies), 10 μl of the CellLight® reagent was added to the culture media of SH-SY5Y cells and incubated O/N at 37°C, then further incubated with 100 nM Alexa647-labeled α-syn sonicated fibrils Cells were imaged live after α-syn seeds addition, using an adapted confocal spinning-disk setup based on an Axiovert 200 body microscope (Zeiss, Germany) with a spinning disk confocal unit (CSU-X1 Yokogawa, Japan) and a back-illuminated EMCCD camera (iXON 897, Andor, UK) on the side port. The temperature was kept at 37°C with constant 5% CO_2_ concentration in a stage-top incubator (Tokai Hit, Japan). Illumination was performed with two different lasers of wavelength 514 nm (Cobolt, Sweden) and 642 nm (Spectra-Physics, USA).

### dSTORM experiments and data analysis

#### Sample preparation for dSTORM

*In vitro* prepared α-syn fibrils, labeled with Alexa 532, were spin coated onto a glass coverslip in 1% poly vinyl alcohol (Sigma Aldrich) and imaged. Imaging was performed in a switching buffer solution: 100 mM mercaptoethylamine (Sigma Aldrich) in PBS (pH 8.0) [24]. On day before imaging, SH-SY5Y were plated at 10^5^ cells on 35 mm ibidi treated glass bottom dishes (ibidi GmbH, Germany) and then incubated with 100 nM final concentration of α-syn seeds labeled with Alexa 532. Cells were fixed in 4% formaldehyde at different incubation times and then imaged in the switching buffer.

#### dSTORM set-up

**Fig 1:**
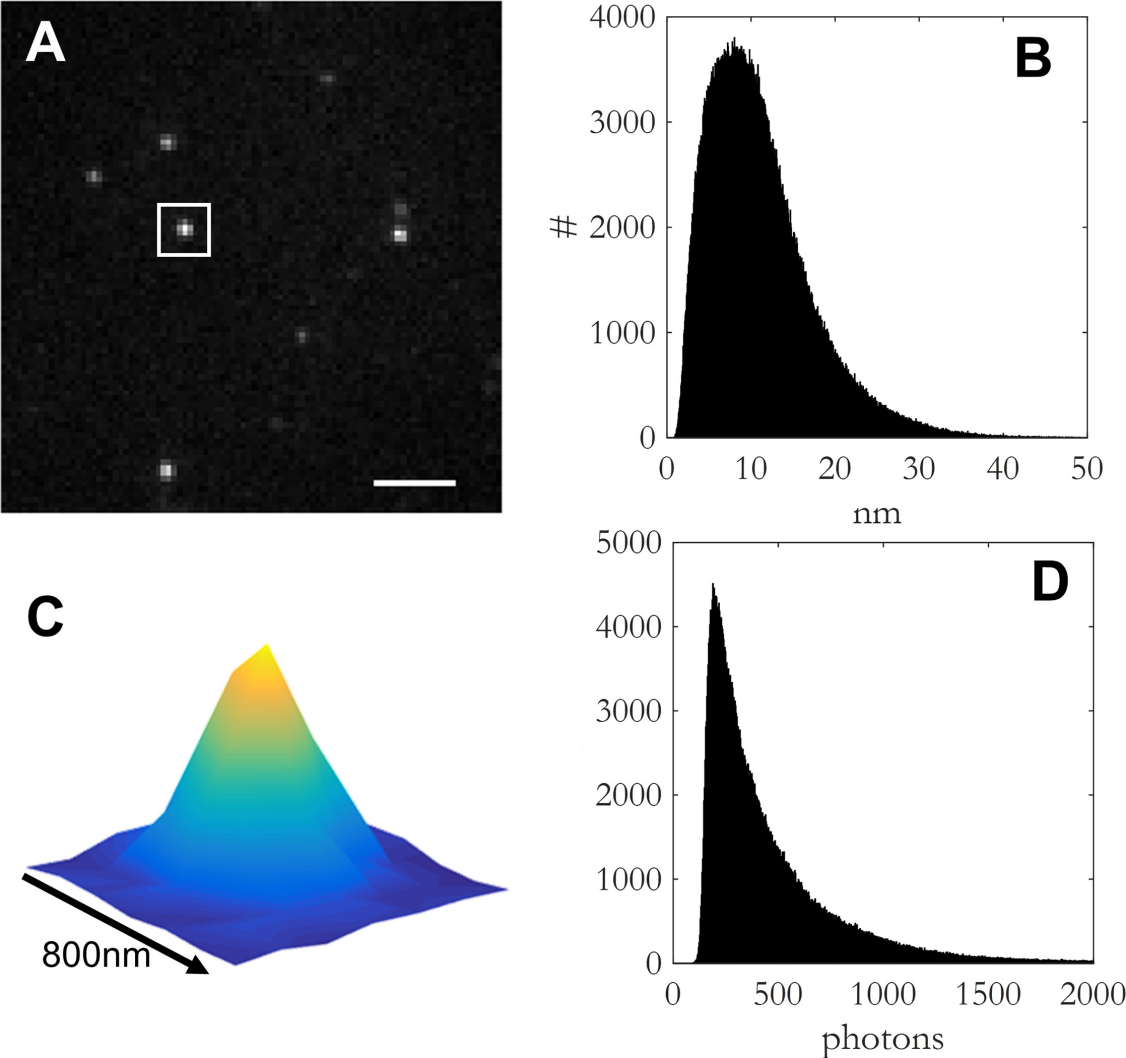
Characteristic properties of the optical setup. (a) Frame with the signal of several Alexa532 molecules. Scale bar = 2μm. (b) Histogram of the sigma of positional accuracy (Mean: 11 nm). (c) Zoom-in of the white square in Fig 1A showing the Gaussian intensity profile. (d) Histogram of the intensity of localizations (Mean: 447 photons).

Super-resolution imaging was performed on a home build wide-field single-molecule setup, based on an Axiovert S100 Zeiss inverted wide-field microscope equipped with a 100× 1.4 NA oil-immersion objective (Zeiss, Germany). The Alexa 532 dye was excited using a 532 nm laser (Cobolt, Sweden). A 405 nm laser (CrystaLaser, USA) was used for photo-switching and to adjust the density of visible fluorophores. The light was reflected into the objective by the dichroic mirror ZT405/532/635rpc (Chroma, USA). The fluorescence light in the detection path was filtered using the emission filter ZET532/633m (Chroma, USA). Excitation intensities were in between 0 and 20 W/cm^2^ at 405 nm and 3 kW/cm^2^ at 532 nm. For each sample, we acquired 10000 single-molecule images with an acquisition time of 10 ms per frame and a frame rate of 87 Hz. The signal of individual dye molecules was captured on a sCMOS Orca Flash 4.0V2 camera (Hamamatsu, Japan) (Fig 1A). The average integrated signal of a single dye molecule was 447 detected photons (Fig 1D), spatially distributed by the 2 dimensional point-spread-function of the microscope of 293 nm FWHM (Fig 1C).

#### Data analysis

The signal from individual fluorophores (Fig 1C) was fitted with a 2 dimensional Gaussian using a custom least-squares algorithm in Matlab [25]. From the fit we determined the location of each molecule to high accuracy of 11 nm on average (Fig 1B). The localization accuracy coincides with the value predicted from the width of the point-spread-function and the detected number of photons, 293 nm / √[447] = 14 nm. Subsequently, the localization data were used to generate super-resolution images. Super-resolution images were obtained by binning localizations into 20×20 nm^2^ bins. For zoom-ins we used probability density maps in which each localization was represented as a normalized Gaussian centered at the position and of width given by the sigma-uncertainty in localization. The pixel size was chosen to represent 1×1 nm^2^.

For analysis of fiber width and length we used line fitting from the raw localization data. We selected a region of interest (ROI) around a fibril. The selected localizations were rotated and subsequently the y-coordinates of the localizations were binned. The full width at half-maximum (FWHM) was calculated from the resulting histogram (see S1 Fig). Fibrils with a FWHM lager than 70 nm were discarded from further analysis. Given that localizations in dSTORM experiments had an average positional accuracy of 11 nm (Fig 1B), leading to an apparent FWHM of 26 nm for any point object. The apparent FWHM of the fiber was therefore de-convolved to give the true fiber width, FWHM_d_ = √[FWHM^2^-(26 nm)^2^].

## Results and Discussion

### Super-resolution imaging of *in vitro* α-syn fibrils

In this study we used direct stochastic reconstruction microscopy (dSTORM) to follow the uptake of α-syn aggregates by the non-transfected SH-SY5Y human neuroblastoma cells. dSTORM has been used in several studies to probe the morphology of protein aggregates such as Aβ-aggregates in Alzheimer’s disease [20, 21], Huntingtin protein aggregates in Huntingtin disease [17, 26] and α-syn amyloid fibrils in Parkinson’s disease (PD) [18]. In relation to earlier studies we here used direct fluorescence labeling of α-syn by switchable fluorophores. Direct labeling is advantageous when compared to other superresolution methods which typically involve immunofluorescence labeling, the latter leading to substantial increase in structure size due to the antibody size (~10 nm) as compared to the small size of fluorescent dyes used in the current study.

**Fig 2:**
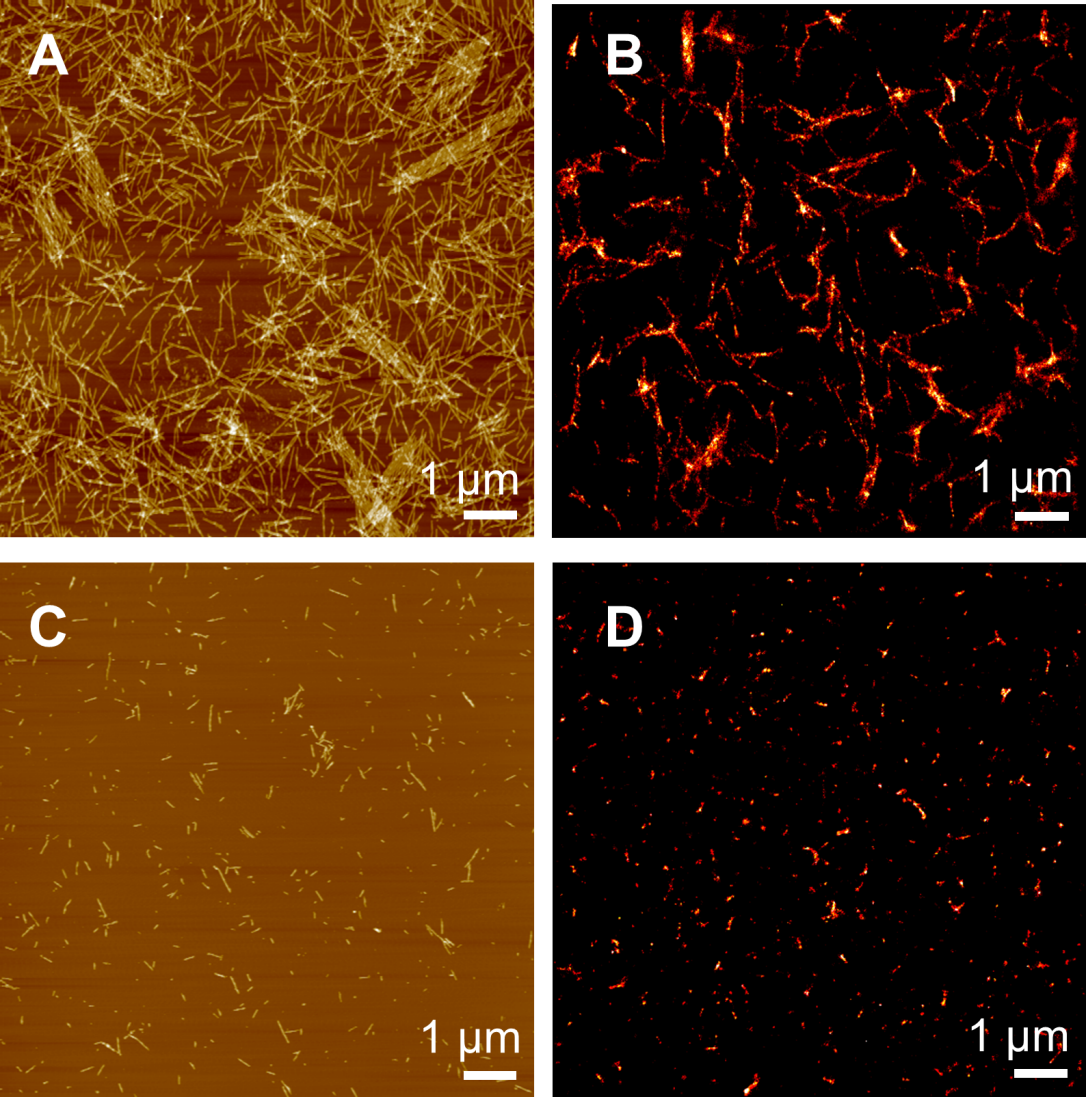
Super-resolution imaging of the *in vitro* prepared α-syn fibrils. (a) AFM and (b) dSTORM images of intact wild-type α-syn fibrils covalently labeled with the NHS derivate of Alexa 532 fluorophore. (c) AFM and (d) dSTORM images of sonicated labeled α-syn fibrils.

We first characterized the properties of α-syn amyloid *in vitro* fibrils prepared in our conditions. Fig 2 shows the morphology of labeled intact α-syn fibrils as obtained by AFM (Fig 2A) and by dSTORM (Fig 2B) when deposited at low concentration onto a flat substrate. Individual fibrils were clearly identified in both methods. The overall images appear very comparable. The length of the fibrils clearly exceeded 1 μm extending to 10 μm and longer. In view of the complex topology of the fibrous network with fiber crossings, a detailed statistical analysis of fiber length was omitted.

**Fig 3:**
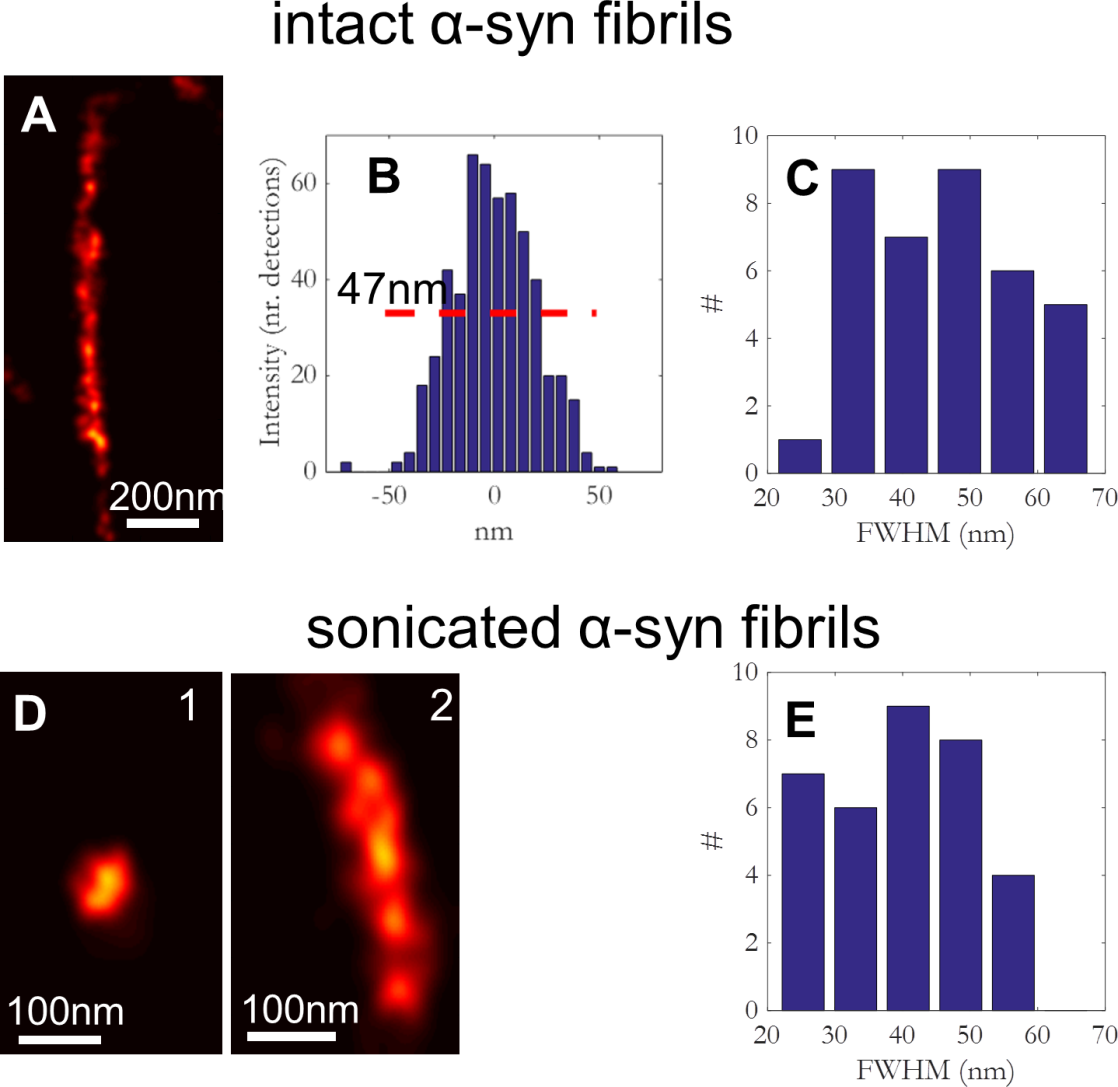
Characterization of different sized α-syn fibrils by dSTORM. (a) Detailed view of an intact α-syn-Alexa532 fibril (the marked fibril from Fig 2A). (b) Histogram corresponding to the localization data (see S1 Fig for details on the method). A FWHM of 47 nm was calculated. (c) Histogram distribution of FWHM for intact α-syn fibrils. A mean diameter of 43 ± 12 nm was calculated from FWHM data (> 25 fibrils). (d) dSTORM images of two different sized sonicated fibrils (the marked sonicated fibrils from Fig 2B). (e) Histogram distribution of FWHM for sonicated α-syn fibrils. The mean diameter of α-syn sonicated fibrils was 42 ± 11 nm.

The apparent width of the fibers was determined from the dSTORM images as detailed in the materials and methods sub-section. A zoom-in of the fibril marked in Fig 2B is shown in Fig 3A. From the cross-sectional average of the intensity with respect to the fibril normal (line in Fig 3A) the apparent width of the fiber was 48 ± 12 nm FWHM. The distribution of apparent widths for 38 fibrils is shown in Fig 3C. The distribution is characterized by a mean of FWHM = 43 ± 12 nm. Deconvolution finally leads to the real fiber widths of FWHMd = 34 ± 12 nm. This result by optical microscopy was compared to results by AFM experiments. In AFM experiments, we used the height information to estimate the diameter of the labeled α−syn fibrils. The mean height was found to be 8 ± 1 nm for the labeled α−syn fibrils (mean ± s.e. from 50 fibers, see S2 Fig). The apparent discrepancy in fiber widths between the two methods probably relates to some additional imperfections due to the angular alignment in the super-resolution imaging, which were not considered in the deconvolution procedure. In any case, fibers appeared as structures of width way below 50 nm.

α-Syn aggregates have been reported to be internalized into a variety of cell types, including neurons [7, 9, 13, 14]. Several studies have shown that exogenously generated α-syn fibrils and not the oligomers, specifically induce intracellular LB-like inclusion formation. It has been also shown that the uptake is more efficient if the fibrils are shorter [9, 11–14]. For this reason, α-syn fibrils were sonicated prior their addition to the cells. The effect of sonication on fibril size is already apparent in Fig 2. Whereas fibril length clearly exceeds 1 μm for the intact fibrils (Fig 2A & 2B), after sonication the length shortened to <1 μm (Fig 2C & 2D) independent of the imaging method used. The lengths of the sonicated fibrils determined by AFM were between 50 and 700 nm. The lengths determined by dSTORM were similar, and in the range between 30 and 650 nm. The smaller length fibrils appear as globular (Fig 3D1) whereas the longer structures as clear fibrils (Fig 3D2). While sonication led to a significant decrease in fibril length the fibril width was unchanged. The distribution of sonicated fibril width, as shown in Fig 3E, is characterized by a mean of 42 ± 11 nm, which leads, after deconvolution, to a real width of FWHM_d_ of 32 ± 11 nm.

### Internalization of extracellular α-syn fibrils into neuroblastoma cells

Uptake and intracellular localization of preformed α-syn aggregates

Having established that we were able to distinguish small sized α-syn aggregates *in vitro*, we moved further to study the fate of the aggregates once they are exogenously added to cells in culture. We investigated the uptake of the small, labeled fibrillar α-syn by the SH-SY5Y human neuroblastoma cells using confocal fluorescence microscopy.

**Fig 4:**
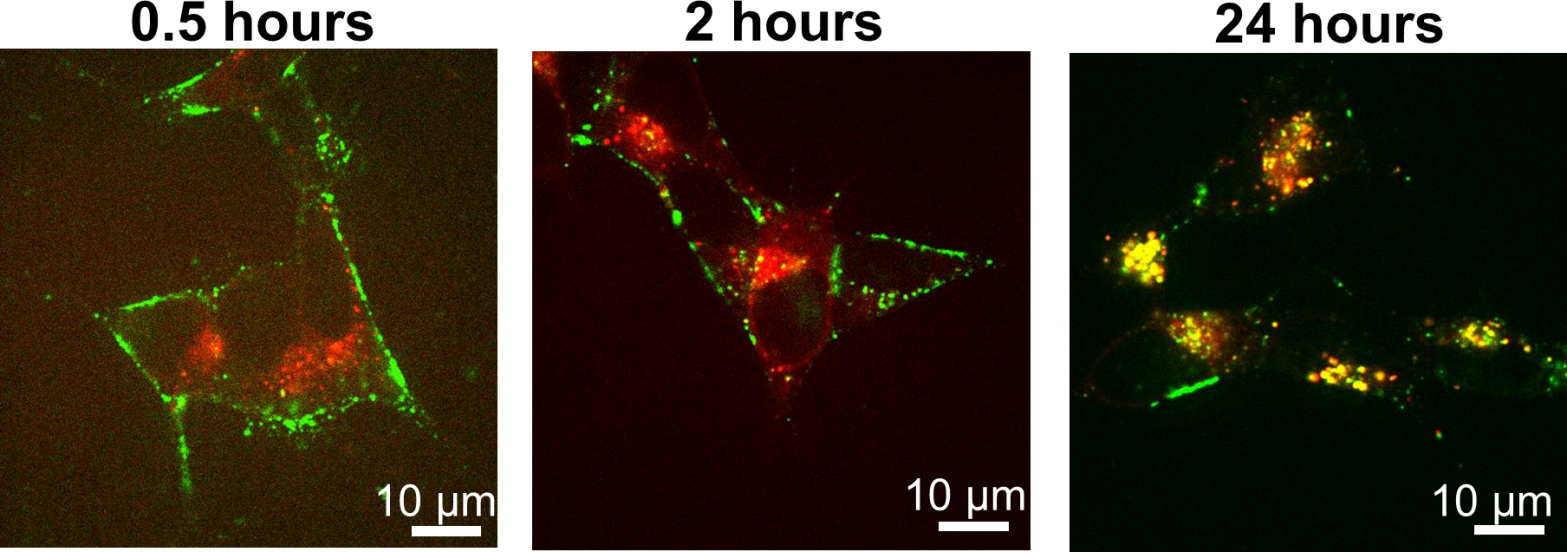
Internalization of α-syn sonicated fibrils in human neuroblastoma cells. Images show co-localization of Alexa 532 labeled α-syn aggregates (green) with LysoTracker Deep Red (red). SH-SY5Y cells were treated with 50 nM LysoTracker Deep Red, then washed, incubated further with Alexa532-labeled α-syn sonicated fibrils and imaged live on a confocal microscope.

The time-course of uptake is seen in Fig 4. Sonicated, labeled seeds were added at a concentration of 100 nM to the culture medium and left during the experiment. Their uptake by SH-SY5Y cells was followed in time using confocal microscopy and dSTORM. In the first 2 hours α-syn aggregates were mostly present at the cell membrane resulting in images that resemble typical images of the cell’s outline (Fig 4 left&middle). After 24 hours Alexa532-syn aggregates disappear from the outer cell membrane and localize as granular intracellular deposits mostly close to the nucleus (Fig 4 right). This observation suggests that fibrils were internalized and probably processed in the endosomal pathway towards perinuclear lysosomes. This interpretation is supported by dual color experiments that showed that the granular deposits (Fig 4 green) colocalized with a marker for acidic organelles. (LysoTracker; Fig 4 red). To further confirm that labeled α−syn aggregates accumulate in lysosomes, we further used CellLight Lysosomes-RFP, BacMam 2.0 (Life Technologies), a fusion construct of Lamp1, to specifically label the lysosomes. Analysis of the SH-SY5Y cells exposed for 24 hours to the Alexa647-labeled α-syn fibrils showed that the aggregates are internalized and they do colocalize with Lamp1, marker protein for lysosomes (S3 Fig). At the same time, we demonstrated that internalization and fate of the aggregates is not influenced by the fluorophores used for labeling. Hence, our data confirm earlier results which suggested that protein aggregates like α-syn are transported towards the lysosomal compartment to be degraded [6, 7].

### Super-resolution imaging of intracellular α-syn aggregates

Subsequently we addressed whether maturation and increase in aggregation of fibrils occurred while transported from the plasma membrane to the endosomes/lysosomes. Whether and how maturation occurs and whether that will lead to some equilibrium distribution of aggregates, monomers and degraded peptide in cells when being continuously exposed to an extracellular concentration is still unclear. One proposed mechanism for maturation, that has been linked to disease, is that fibrils further aggregate inside the acidic endocytic vesicles as the combination of low pH and high effective concentration are favorable conditions for α-syn aggregation [27]. Further it is unclear whether such maturation would lead to the formation of long α-syn fibrils in the cytoplasm of the cells.

**Fig 5:**
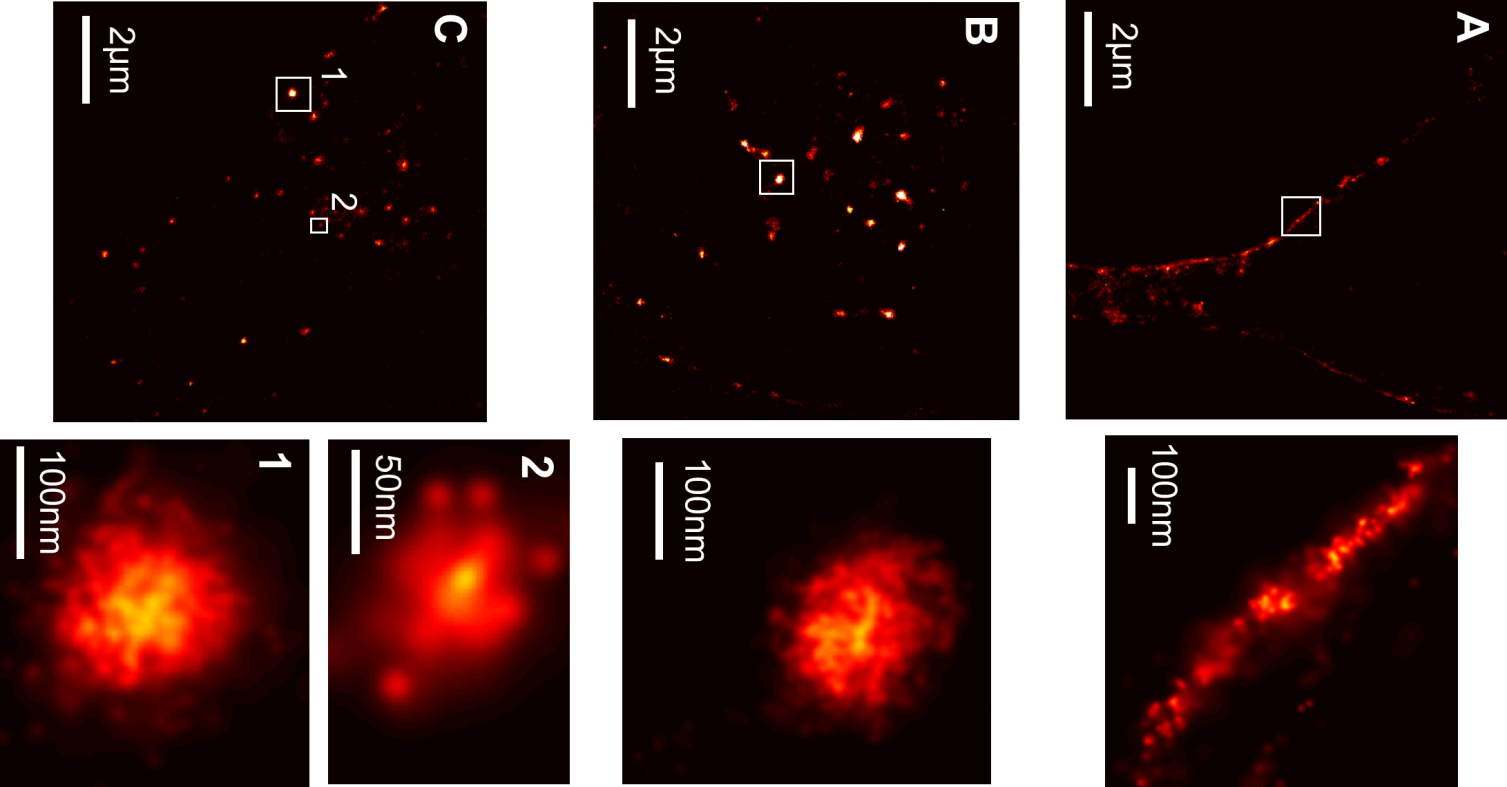
Super-resolution images of internalized α-syn aggregates in endosomal vesicles in time. (a) dSTORM image of a cell treated for half an hour with α-syn -Alexa532 aggregates. A detailed view of the aggregates in the cell membrane is shown below a). (b) After 2 hours of incubation, α-syn aggregates are internalized in vesicles. Detailed view of the aggregates in a vesicle shown in the image below b). (c) Internalized α-syn aggregates after 24 hours of incubation, with two different sized clusters highlighted bellow image c).

To address these questions, we used the super-resolution capability of dSTORM to probe directly the morphology of α-syn aggregates while entering SH-SY5Y cells (Fig 5). The dSTORM images, like the confocal images in Fig 4, showed that α-syn aggregates initially associated with, and accumulated at the plasma membrane (Fig 5A). In the course of time cells took up the fibrils and accumulated them in endocytic vesicles (Fig 5B & 5C). Inside the vesicles the aggregates appeared tightly packed forming bigger clusters. The typical size of those intracellular globular structures stretched from 30–150 nm. It is worth mentioning that the small-sized fibrils did not completely lose their morphology at the length scale of our resolution even after 72 hours (Fig 5B & 5C). Endosomes and lysosomes are typically 50–400 nm in diameter and are distributed uniformly throughout the cytosol [28]. Hence, the size of the clusters, as determined by live-cell dSTORM, falls into the small size region expected for endosomes and lysosomes. We considered the possibility of lysosomal membrane decoration by α-syn. However this would present as ring-like structures in our super-resolution images of vesicles larger than then twice FWHM of ours localizations (52nm). This has not been observed in our data, even for vesicles >100nm.

**Fig 6:**
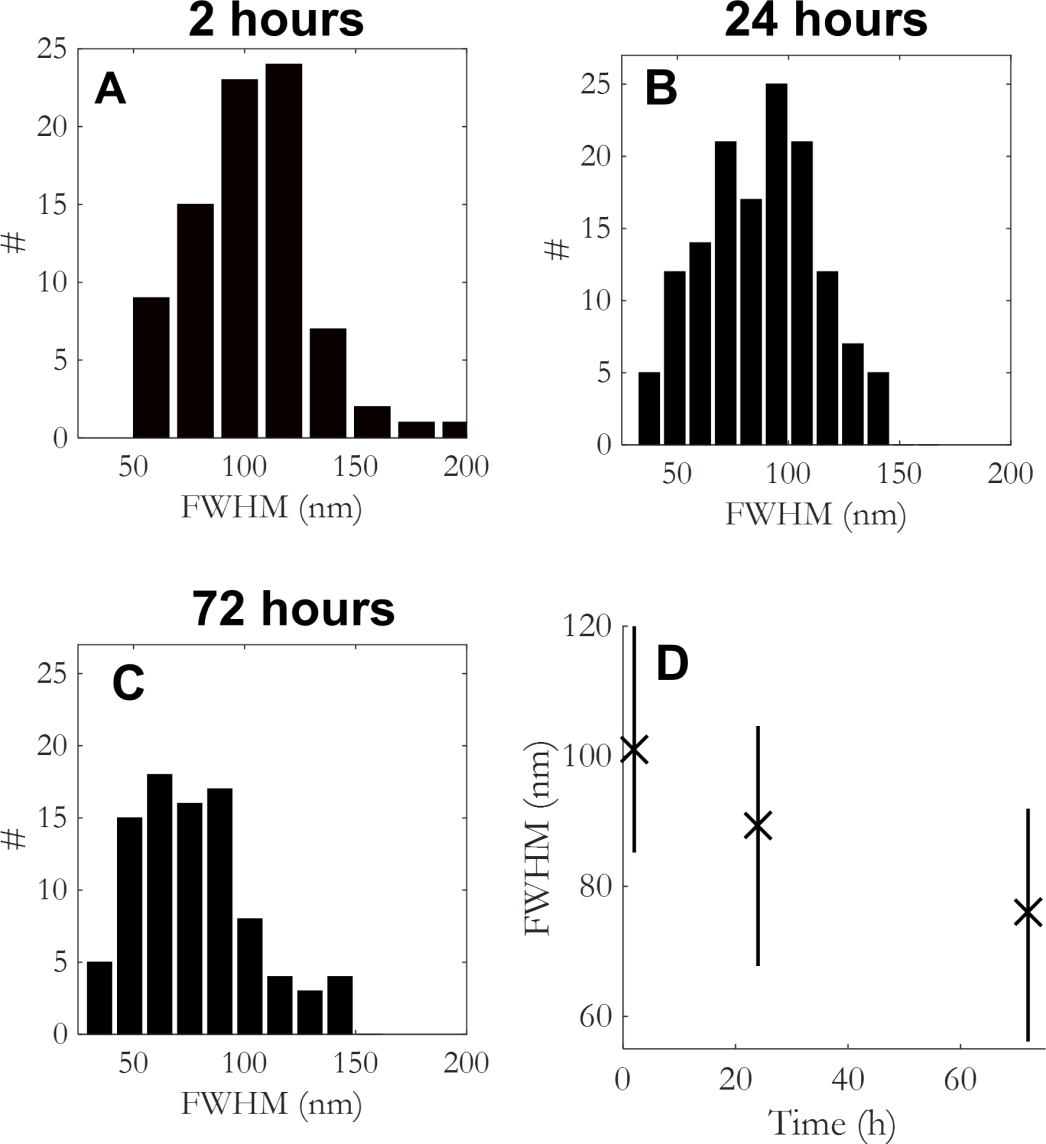
Size distribution of α-syn aggregates in endosomal vesicles in time. (a)-(c) Histogram of FWHM of intracellular α-syn clusters in time. (ANOVA significance levels: (a)-(b): 10^−4^; (b)-(c):5×10^−3^; ((a)-(c):10^−7^). (d) A decrease in α-syn cluster size is observed in the mean average FWHM of α-syn clusters in time (median and 50% interval).

The size of the clusters appeared to decrease as proteins moved through the endosomal pathway towards lysosomes (Fig 6). The size decreased from 104 ± 32 nm at 2 h after incubation to 78 ± 28 nm after 72 h. This significant decrease might be interpreted as onset of lysosomal breakdown. It should be stressed that we did not observe maturation and formation of α-syn fibrils in the cytoplasm over a period of 3 days. It is important to note that, with the exception of Lewy bodies [9, 29] there is so-far no clear evidence for the presence of linear α-syn amyloid fibrils within mammalian cells. Similar studies are needed in primary cultured neurons where it has been shown that formation of α-syn pathology is more efficient than in immortallized neuroblastoma cells, especially in mature neurons with higher levels of α-syn expression at presynaptic terminals [9].

## Conclusions

In conclusion, we demonstrated that using dSTORM, it is possible to follow noninvasively the endocytotic uptake of extracellularly added amyloid aggregates and get morphological details of these in the vesicular compartments. Our study provides also additional evidence in favor of lysosomal degradation pathway for removal of extracellular α-syn aggregates. Cells internalize extracellular small sized fibrils (<1 μm length). Subsequently the aggregates accumulate in endocytic vesicles and are trafficked towards lysosomes. Fibrils keep their morphology and do not further mature but rather shortened possibly due to lysosomal breakdown as they move through endosomal pathway. As lysosomal malfunction has been linked to neurodegeneration and age-related neurodegenerative disorders [30, 31], enhancing lysosomal function may be a potential therapeutic strategy for prevention or treatment of PD. Since our study did not focus on the effect of longer (>1 μm) fibers on cellular processes and cell viability, their potential impact of long fibers in disease should not be overlooked.

## Supporting Information

S1 Fig Method for width determination of fibrils. (a) N = 526 localizations of the single fibril seen in Fig 3A (scale bar = 50nm). (b) Locations are rotated so the angle of a linear fit is 0 (red line). (c) Y- coordinates are binned into √N bins. FWHM is determined from linear interpolation of the histogram to be 47 nm. (red dashed line).
(PDF)

S2 Fig Height distribution of fibrils. Height distribution of fibrils as determined by atomic force microscopy.
(PDF)

S3 Fig Colocalization α-syn with lysosomes. Co-localization of Alexa 647labeled α-syn sonicated fibrils (red) with the lysosomes labeled with CellLight Lysosomes-RFP (green) after O/N incubation of the cells with the CellLight reagent, followed by 24 hours of incubation with 100 nM Alexa 647-α-syn fibrils.
(PDF)

S1 File Essential Data File. Data represented in Figs 1, 3, 5, 6, S1 and S2.
(ZIP)

S2 File Essential Data Fig 2B. Data represented in Fig 2B.
(ZIP)

S3 File Essential Data Fig 2D. Data represented in Fig 2D.
(ZIP)
