# Supplementary figures and images for: Direct Observation of α-Synuclein Amyloid Aggregates in Endocytic Vesicles of Neuroblastoma Cells
Source: PLoS One. 2016 Apr 22;11(4):e0153020. doi: 10.1371/journal.pone.0153020 (PMC4841506; doi:10.1371/journal.pone.0153020)

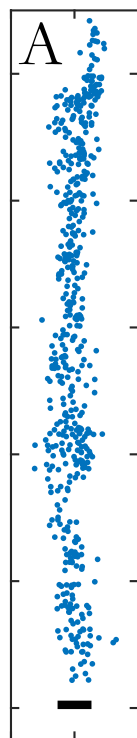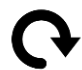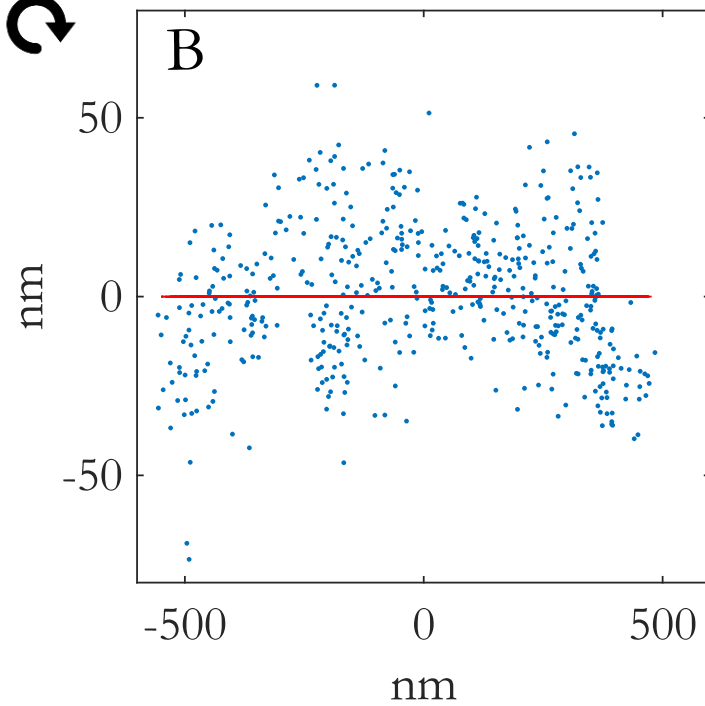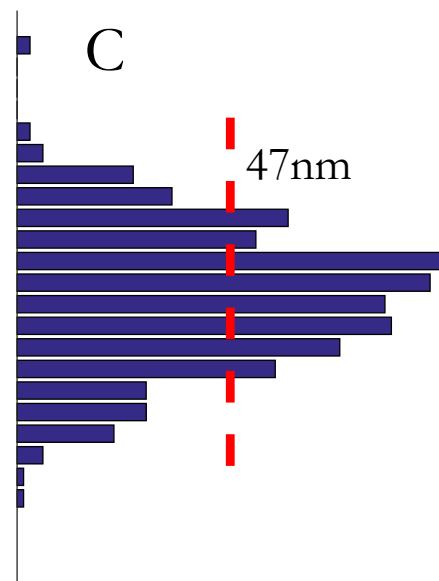

Supplement: S1 Fig — (a) N = 526 localizations of the single fibril seen in Fig 3A (scale bar = 50nm). (b) Locations are rotated so the angle of a linear fit is 0 (red line). (c) Y- coordinates are binned into √N bins. FWHM is determined from linear interpolation of the histogram to be 47 nm. (red dashed line). (PDF) [file pone.0153020.s001.pdf]

**Figure S2.**

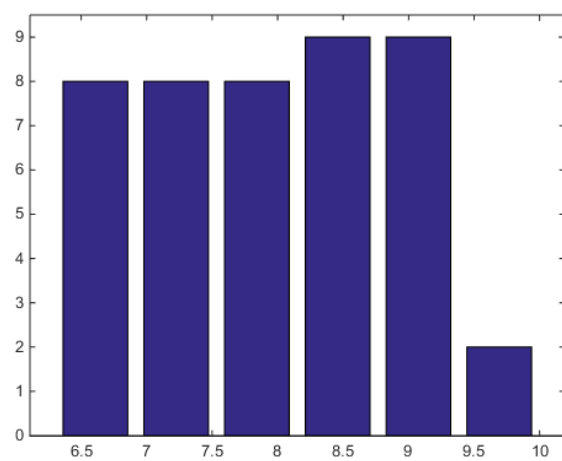

Supplement: S2 Fig — Height distribution of fibrils as determined by atomic force microscopy. (PDF) [file pone.0153020.s002.pdf]

Figure S3.

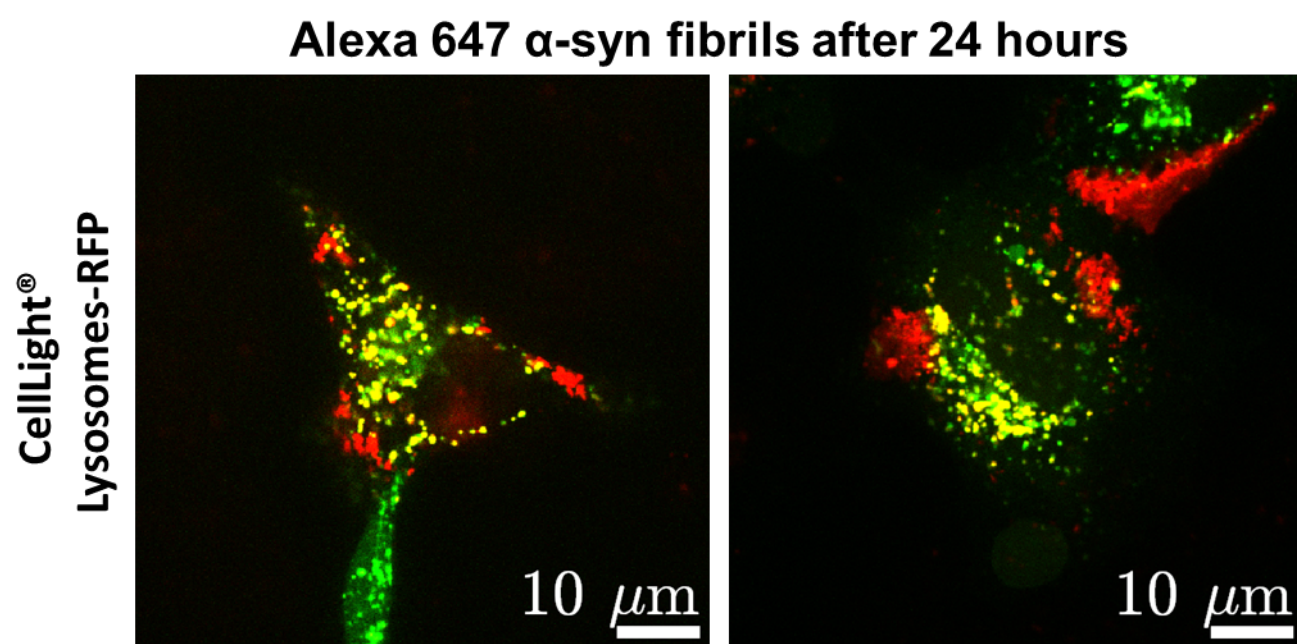

Supplement: S3 Fig — Co-localization of Alexa 647labeled α-syn sonicated fibrils (red) with the lysosomes labeled with CellLight Lysosomes-RFP (green) after O/N incubation of the cells with the CellLight reagent, followed by 24 hours of incubation with 100 nM Alexa 647-α-syn fibrils. (PDF) [file pone.0153020.s003.pdf]
